# Machine learning approaches to predict lupus disease activity from gene expression data

**DOI:** 10.1038/s41598-019-45989-0

**Published:** 2019-07-03

**Authors:** Brian Kegerreis, Michelle D. Catalina, Prathyusha Bachali, Nicholas S. Geraci, Adam C. Labonte, Chen Zeng, Nathaniel Stearrett, Keith A. Crandall, Peter E. Lipsky, Amrie C. Grammer

**Affiliations:** 1 RILITE Research Institute and AMPEL BioSolutions, 250 W Main St, Ste 300, Charlottesville, VA 22902 USA; 2 Department of Physics, George Washington University, Washington, DC 20052 USA; 3 Computational Biology Institute, Milken Institute School of Public Health, George Washington University, Washington, DC 20052 USA

**Keywords:** Machine learning, Autoimmunity, Systemic lupus erythematosus

## Abstract

The integration of gene expression data to predict systemic lupus erythematosus (SLE) disease activity is a significant challenge because of the high degree of heterogeneity among patients and study cohorts, especially those collected on different microarray platforms. Here we deployed machine learning approaches to integrate gene expression data from three SLE data sets and used it to classify patients as having active or inactive disease as characterized by standard clinical composite outcome measures. Both raw whole blood gene expression data and informative gene modules generated by Weighted Gene Co-expression Network Analysis from purified leukocyte populations were employed with various classification algorithms. Classifiers were evaluated by 10-fold cross-validation across three combined data sets or by training and testing in independent data sets, the latter of which amplified the effects of technical variation. A random forest classifier achieved a peak classification accuracy of 83 percent under 10-fold cross-validation, but its performance could be severely affected by technical variation among data sets. The use of gene modules rather than raw gene expression was more robust, achieving classification accuracies of approximately 70 percent regardless of how the training and testing sets were formed. Fine-tuning the algorithms and parameter sets may generate sufficient accuracy to be informative as a standalone estimate of disease activity.

## Introduction

SLE is a complex, multisystem autoimmune disease that continues to be a major diagnostic as well as therapeutic challenge. There are no definitive, specific diagnostic tools available to determine whether a patient has SLE, and diagnostic approaches in SLE have not changed in decades. Physicians still rely on clinical evaluation and a few laboratory tests, including measurement of autoantibodies and complement levels. Despite the wealth of genetic, epigenetic, and gene expression data that has emerged in the past few years at both the patient and cellular levels, none has been integrated to produce a predictive tool that can be used to evaluate an individual SLE patient.

In SLE, defects in central and peripheral tolerance allow for activation of self-reactive B cell clones and differentiation into plasmablasts/plasma cells (PCs) that secrete autoantibodies, which in turn mediate tissue damage^1,2^. Genome wide association studies (GWAS) have identified numerous polymorphisms in regions encoding genes or regulatory regions that could influence B cell function^3^, suggesting that a general state of B cell hyper-responsiveness could contribute to SLE pathogenesis. Autoantibody-containing immune complexes stimulate production of type 1 interferon, a hallmark of infection that is also observed in SLE patients, regardless of disease activity^4,5^. In addition to B cells and PCs^6^, various T cell populations also exert differential effects on SLE pathogenesis. T follicular helper cell subsets contribute to B cell activation and differentiation, and abnormal T cell receptor signaling is also thought to lead to hyper-responsive autoreactive T cell activity^7–9^. Furthermore, defects in regulatory T cells, partially secondary to deficient IL-2 production, result in faulty modulation of immune activity and inflammation^8,9^.

Myeloid cells (MC) also play a role in SLE pathogenesis^10^. Factors present in the local microenvironment can cause macrophages (Mϕ) to undergo extreme changes in transcriptional regulation in a process called Mϕ polarization^11–13^. Overabundance of proinflammatory M1 Mϕ and decreased expression of markers for anti-inflammatory M2 Mϕ are detected in both lupus-prone mice and SLE patients^14,15^, and therapeutic stimulation of M2 polarization significantly decreases disease severity in murine SLE^16^. Experimental intervention in M2 polarization as well as microRNA array profiling suggest that abnormalities in M2 Mϕ may contribute to SLE severity^15,17^. Low-density granulocytes (LDGs) are abnormal neutrophil-like cells that appear in the blood of lupus patients as well as in many other disease states^18–23^. Although their involvement in SLE has not been studied as extensively as that of other cell types, LDGs have already been linked to kidney disease, vascular disease, and other manifestations in lupus patients^24–29^.

To date, however, it has been difficult to relate gene expression profiles to SLE disease activity successfully. Numerous groups have attempted to characterize SLE patients by gene expression. For example, Jourde-Chiche *et al*. reported a discrete group of differentially expressed genes that might be found in subjects with SLE renal disease^28^, and Banchereau *et al*. extensively analyzed pediatric lupus samples and attempted to associate modules of expressed genes with disease manifestations in children^30^. Despite these advances, gene expression data has yet to provide an approach with sufficient predictive value to utilize in decision making about individual subjects with SLE. Furthermore, no cellular phenotype has been independently verified to be able to distinguish a patient with active SLE from one with inactive disease. This distinction is critical both for patient evaluation and for clinical trials, as most SLE trials are aimed at controlling disease activity.

Therefore, in order to advance personalized treatment of SLE patients, the use of big data analytical techniques, including machine learning, can be useful to understand the relationships between cell subsets, gene expression, and disease activity. Machine learning describes a wide range of computational methods which allow researchers to harness complex data and develop self-trained strategies to predict the characteristics of new samples, such as whether a given SLE patient has active or inactive disease. Machine learning techniques have already been leveraged in lupus to characterize disease risk and identify new biomarkers based on genotypic data or urine tests^31,32^. When applied to high-throughput transcriptomic data, machine learning algorithms could potentially be used to identify the gene expression features with the most utility to identify subjects with higher degrees of disease activity and may also provide insights into disease pathogenesis.

To address this possibility, we used conventional bioinformatics methods in conjunction with unsupervised and supervised machine learning techniques to: (1) test the potential of raw gene expression data and modules of genes to classify subjects with active and inactive SLE, (2) determine the optimum classifier or classifiers, and (3) understand the combinations of variables that best facilitate classification.

## Results

### Gene expression and SLE disease activity

Before employing machine learning techniques, it was necessary to first assess whether conventional bioinformatics approaches could accurately separate active SLE patient samples from those obtained from inactive patients. First, three whole blood (WB) data sets (Table 1) were filtered to include only those genes which passed quality control and filtering in all three studies. Differential expression (DE) analysis of active versus inactive patient samples with the remaining filtered 7,848 genes revealed major differences among data sets and considerable heterogeneity within data sets. GSE39088 had only 176 DE genes with a false discovery rate (FDR) less than 0.2 and none with FDR < 0.05; GSE45291 had 5850 DE genes with FDR < 0.2 and 4837 with FDR < 0.05; GSE49454 had 1710 DE genes with FDR < 0.2 and 72 with FDR < 0.05 (Supplementary Data S1). Hierarchical clustering was carried out on each study with all genes, DE genes with FDR < 0.2, and DE genes with FDR < 0.05 to determine whether active and inactive patients would separate into two clusters. The Adjusted Rand Index (ARI) was used to compare these clusterings to the known status of the patients. When using all genes, all three studies had ARIs near zero, indicating that clustering separated active and inactive patients no better than random chance (Table 2). GSE39088 and GSE49454 showed only mild improvement after filtering genes, whereas GSE45291 attained an ARI of 0.94 when using genes with FDR < 0.05.

**Table 1:** Data sources for active (SLEDAI ≥ 6) and inactive (SLEDAI < 6) SLE WB gene expression.

Accession	Microarray Platform	N Active	N Inactive	SLEDAI Range	SLEDAI Mean (SD)
GSE39088	GPL570 (Affymetrix HG-U133 + 2.0)	24	13	2–12	6.8 (2.7)
GSE45291	GPL13158 (Affymetrix HG-U133 + PM)	35	35	0–11	4.3 (3.5)
GSE49454	GPL10558 (Illumina HumanHT-12 v4.0)	23	26	0–26	7.7 (7.2)

Data sets are listed by Gene Expression Omnibus (GEO) accession numbers. N Active/Inactive: number of active/inactive patients in data set. Range, mean, and standard deviation of SLEDAI values in each data set are provided.

**Table 2:** Adjusted Rand Index of Unsupervised Hierarchical Clustering Compared to Known Disease Activity.

	Adjusted Rand Index
GSE39088	−0.04
GSE39088; FDR < 0.2	0.19
GSE39088; FDR < 0.05	N/A
GSE45291	0.03
GSE45291; FDR < 0.2	−0.01
GSE45291; FDR < 0.05	0.94
GSE49454	0.04
GSE49454; FDR < 0.2	0.14
GSE49454; FDR < 0.05	0.14
All Studies	0.03
All Studies; Three Consistent DE Genes	0.05

Data sets are listed by GEO accession numbers. GSE39088 had no genes with FDR < 0.05. The “Three Consistent DE Genes” are DNAJC13, IRF4, and RPL22.

**Figure 1:**
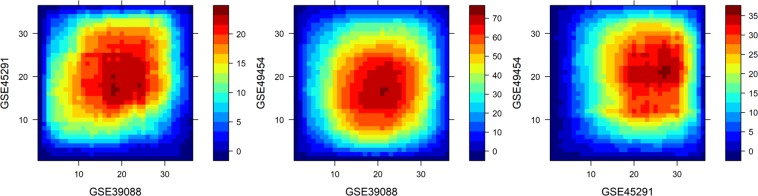
Heatmaps of −log10(overlap p values) from RRHO. Strongest overlaps near the center of each plot indicate weak agreement among the most significantly upregulated and downregulated genes from each data set. Strong agreement between data sets should form a diagonal from the bottom-left corner to the top-right corner.

Next, the lists of genes were compared for commonalities. Out of 6,440 unique DE genes from the three studies, 5,170 genes were unique to one study, 1,234 were shared by two studies, and 36 were shared by all three studies. Of these 36 genes, only three had consistent fold changes across all studies (DNAJC13 and IRF4 upregulated; RPL22 downregulated). Rank-rank Hypergeometric Overlap (RRHO) was next applied as a threshold-free comparison of the studies^33^. All genes that were tested for differential expression were sorted by FDR from most significantly overexpressed to most significantly underexpressed and broken into 36 groups of 218 genes each. Among the three studies, the ranked gene lists failed to demonstrate significant overlap of the most overexpressed and underexpressed genes (Fig. 1). The three data sets were comprised of different patient populations and were collected on different microarray platforms (Table 1); still, the heterogeneity is striking. The lack of commonality among the genes most descriptive of active and inactive patients in each data set casts doubt on whether active and inactive patients from different data sets will separate cleanly.

**Figure 2:**
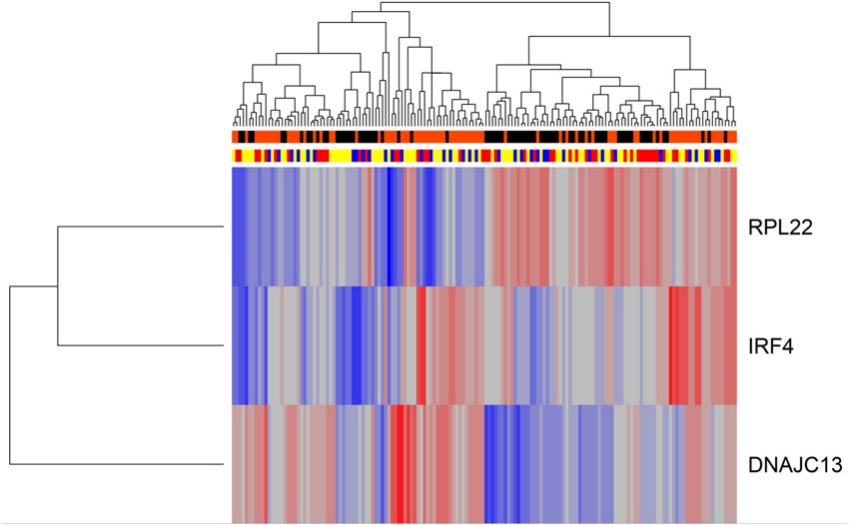
Clustering all three studies on three consistent DE genes. DNAJC13, IRF4, and RPL22 were consistently differentially expressed in each study yet fail to fully separate active from inactive patients. Orange bars denote active patients; black bars denote inactive patients. Blue, yellow, and red bars denote patients from GSE39088, GSE45291, and GSE49454, respectively.

Patients from each study were then joined to evaluate whether unsupervised techniques would separate active patients from inactive patients. Expression profiles from each study were first normalized to have zero mean and unit variance. Figure 2 shows that even these three genes (DNAJC13, IRF4, and RPL22) failed to separate active patients from inactive patients precisely. Hierarchical clustering on all genes had an ARI of 0.03 when compared to the known status of the patients, and clustering on the three consistent DE genes shared among the studies (DNAJC13, IRF4, and RPL22) had an ARI of 0.05 (Table 2). If gene expression has the potential to identify active SLE patients robustly, conventional bioinformatics techniques failed to harness that, highlighting the need for more advanced algorithms.

We hypothesized that patterns of enrichment of Weighted Gene Co-expression Network Analysis (WGCNA) modules derived from isolated cell populations that are correlated to the SLEDAI SLE disease activity index might be more useful than gene expression across studies to identify active versus inactive lupus patients. To characterize the relationships between SLE gene signatures from various peripheral cellular subsets and disease activity, WGCNA was used to generate co-expression gene modules from purified populations of cells from subjects with active SLE, which could subsequently be tested for enrichment in whole blood of other SLE subjects. WGCNA analysis of leukocyte subsets resulted in several gene modules with significant Pearson correlations to SLEDAI (all |r| > 0.47, p < 0.05). CD4, CD14, CD19, and CD33 cells yielded 3, 6, 8, and 4 modules significantly correlated to disease activity, respectively (Table 3). Two low-density granulocyte (LDG) modules were created by performing WGCNA analysis of LDGs along with either SLE neutrophils or HC neutrophils and merging the modules most strongly expressed by LDGs. Two plasma cell (PC) modules were created by using the most increased and decreased transcripts of isolated SLE plasma cells compared to SLE naïve and memory B cells^2^.

**Table 3:** Cell module correlations to disease activity and functional analysis.

Cell Type	Module Name	Module Size	Correlation with SLEDAI	Top GO Biological Process	Top BIG-C Category
CD4	Floralwhite	237	0.81	type I interferon signaling pathway	Interferon-Stimulated-Genes
	Turquoise	805	0.50	positive reg of ubiquitin-protein ligase	Proteasome
	Orangered4	237	−0.77	translational initiation	mRNA-Translation
CD14	Plum1	247	0.47	ubiquitin-dependent protein catabolic process	mRNA-Translation
	Yellow	356	0.65	type I interferon signaling pathway	Interferon-Stimulated-Genes
	Greenyellow	89	−0.49	transcription from RNA polymerase II promoter	General-Transcription
	Pink	261	−0.77	protein phosphorylation	Endosome-and-Vesicles
	Purple	124	−0.66	inositol phosphate metabolic process	Fatty-Acid-Biosynthesis
	Sienna3	222	−0.64	translational initiation	mRNA-Translation
CD19	Darkolivegreen	591	0.78	cell division	Proteasome
	Greenyellow	251	0.66	Notch signaling pathway	mRNA-Translation
	Steelblue	146	0.65	gluconeogenesis	Glycolysis-Gluconeogenesis
	Turquoise	572	0.50	ER to Golgi vesicle-mediated transport	Unfolded-Protein-and-Stress
	Violet	566	0.61	mitochondrial respiratory chain complex I	Interferon-Stimulated-Genes
	Brown	620	−0.62	regulation of transcription, DNA-templated	Chromatin-Remodeling
	Green	541	−0.49	transcription, DNA-templated	Transcription-Factors
	Skyblue	756	−0.74	viral transcription	mRNA-Translation
CD33	Royalblue	94	0.60	positive reg of cytosolic calcium ions	Transposon-Control
	Sienna3	133	0.76	type I interferon signaling pathway	Interferon-Stimulated-Genes
	Violet	177	0.79	defense response to virus	Interferon-Stimulated-Genes
	Darkmagenta	273	−0.49	ubiquinone biosynthetic process	MHC-Class-TWO
LDG^+^	LDG_A	334	0.79	platelet degranulation	Cytoskeleton
	LDG_B	92	0.81	regulation of transcription	Secreted-Immune
	LDG_C	82	−0.39	viral process	Nucleus-and-Nucleolus
PC*	PC_Up	423	N/A	protein N-linked glycosylation	Endoplasmic-Reticulum
	PC_Down	183	N/A	antigen processing and presentation MHC II	MHC-Class-TWO

Information on cell modules including number of genes, Pearson correlation coefficient to SLEDAI, and functional analysis. ^+^LDG modules were generated by WGCNA meta-analysis, and r values indicate separation from control and SLE neutrophils as SLEDAI was unavailable. *PC modules are based solely on differential expression. LDG: low-density granulocyte; PC: plasma cell.

Gene Ontology (GO) analysis of the genes within each module showed that some processes, such as those related to interferon signaling, RNA transcription, and protein translation, were shared among cell types, whereas other processes were unique to certain cell types (Table 3) and might be used to classify patients more effectively. The genes in each module are available in (Supplementary Data S2).

**Figure 3:**
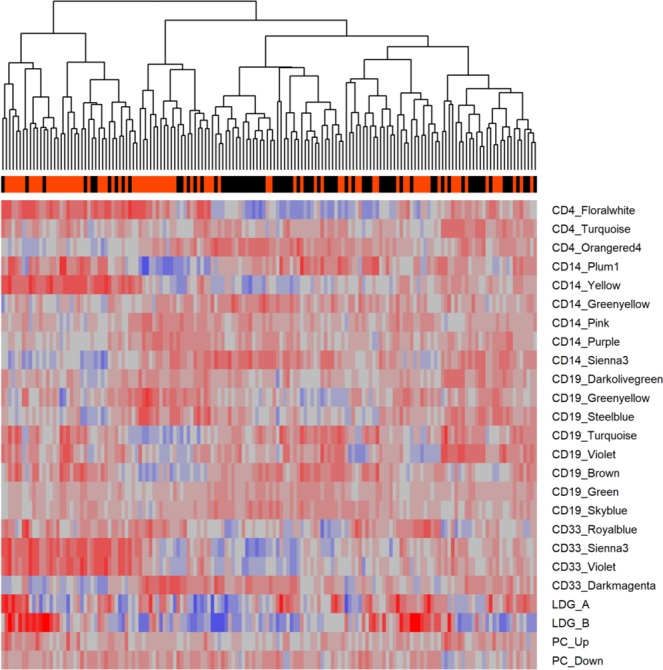
Cellular gene modules provide the basis for machine learning predictions of SLE activity. GSVA was performed on three SLE WB datasets using 25 WGCNA modules made from purified SLE cells with correlation or published relationship to SLEDAI (See Table 2). Orange: active patient; black: inactive patient. LDG: low-density granulocyte; PC: plasma cell.

**Figure 4:**
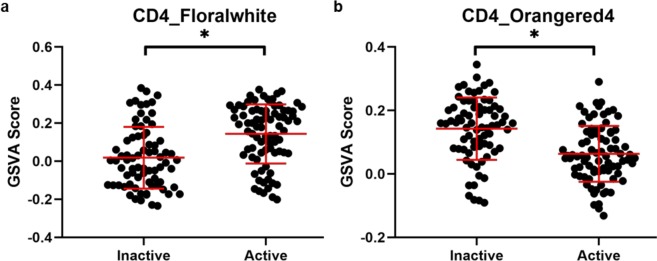
Individual WGCNA modules are ineffective at separating active and inactive SLE subjects. GSVA enrichment scores for (**a**) CD4_Floralwhite and (**b**) CD4_Orangered4 in SLE WB are unable to fully separate active patients from inactive patients. Asterisks denote significant differences by Welch’s *t*-test. Error bars indicate mean ± standard deviation.

To characterize the relationships between SLE gene modules from cell subsets and disease activity in greater detail, Gene Set Variation Analysis (GSVA) enrichment was carried out using the 25 cell-specific gene modules (Fig. 3). Of the 25 cell-specific modules, 12 had enrichment scores with significant Spearman correlations to SLEDAI (p < 0.05), and 14 had enrichment scores with significant differences between active and inactive patients (Welch’s *t*-test, p < 0.05) (Table 4). Notably, each cell type produced at least one module with a significant correlation to SLEDAI in WB and at least one module with a significant difference in enrichment scores between active and inactive patients, demonstrating a relationship between disease activity in specific cellular subsets and overall disease activity in WB. However, the Spearman’s rho values ranged from −0.40 to +0.36, suggesting that no one module had substantial predictive value. Furthermore, the effect sizes as measured by Cohen’s *d* when testing active versus inactive enrichment scores ranged from −0.85 to +0.79. The CD4 Floralwhite and Orangered4 modules, which had the largest positive and negative effect sizes, respectively, showed a high degree of overlap in the enrichment scores of active and inactive patients (Fig. 4).

**Table 4:** Assessment of WGCNA module relationships with SLE disease activity in WB.

	Spearman correlation to SLEDAI		Active vs. Inactive t-test		
	rho	p value	t statistic	p value	d
CD4_Floralwhite	**0.360**	3.90E-06	**4.90**	2.40E-06	0.788
CD4_Turquoise	−0.044	0.587	−0.93	0.352	−0.149
CD4_Orangered4	**−0.400**	2.21E-07	**−5.29**	4.35E-07	−0.853
CD14_Plum1	0.010	0.904	−0.35	0.729	−0.054
CD14_Yellow	**0.356**	4.93E-06	**4.76**	4.44E-06	0.761
CD14_Greenyellow	−0.132	0.100	**−2.10**	0.037	−0.339
CD14_Pink	−0.026	0.751	0.13	0.894	0.021
CD14_Purple	−0.149	0.064	−1.65	0.101	−0.263
CD14_Sienna3	**−0.368**	2.27E-06	**−4.99**	1.62E-06	−0.799
CD19_Darkolivegreen	0.020	0.809	−0.06	0.953	−0.010
CD19_Greenyellow	**0.192**	0.016	**2.55**	0.012	0.403
CD19_Steelblue	0.016	0.838	0.55	0.580	0.089
CD19_Turquoise	−0.069	0.393	−0.84	0.403	−0.132
CD19_Violet	−0.087	0.282	−1.48	0.141	−0.236
CD19_Brown	−0.050	0.537	−1.04	0.301	−0.164
CD19_Green	−0.150	0.062	**−2.07**	0.040	−0.330
CD19_Skyblue	**−0.205**	0.010	**−2.35**	0.020	−0.378
CD33_Royalblue	**0.308**	8.99E-05	**3.99**	1.03E-04	0.637
CD33_Sienna3	**0.362**	3.41E-06	**4.69**	6.15E-06	0.753
CD33_Violet	**0.322**	4.15E-05	**4.35**	2.46E-05	0.696
CD33_Darkmagenta	**−0.216**	6.74E-03	**−2.34**	0.021	−0.369
LDG_A	−0.044	0.588	−0.25	0.802	−0.040
LDG_B	**0.220**	5.71E-03	**2.37**	0.019	0.377
PC_Up	**0.262**	9.75E-04	**3.21**	1.61E-03	0.508
PC_Down	0.022	0.781	0.80	0.426	0.129

Statistics on WGCNA module relationships with SLEDAI and active disease. Correlation to SLEDAI was done by Spearman rank correlation, and the relationship with active versus inactive disease was assessed by Welch’s unequal variances *t*-test and Cohen’s *d*. Significant results are bolded (p < 0.05). LDG: low-density granulocyte; PC: plasma cell.

Analysis of individual disease activity-associated peripheral cellular subset gene modules was not sufficient to predict disease activity in unrelated WB data sets, since no single module from any cell type was able to separate active from inactive SLE patients (Fig. 3). The results emphasized the need for more advanced analysis to employ gene expression analysis to predict disease activity.

### Machine learning and disease activity

**Figure 5:**
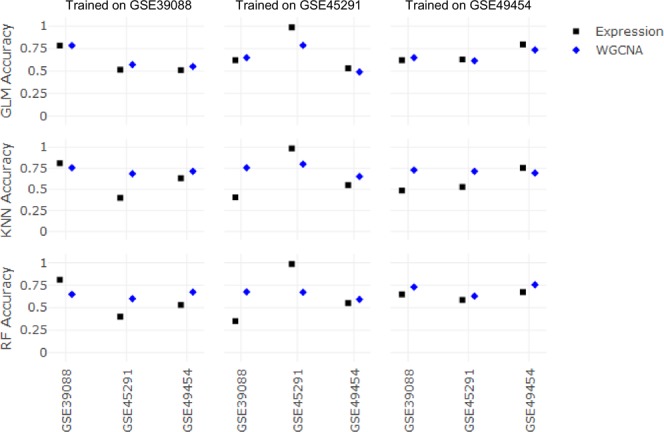
Performance of machine learning classifiers across three independent data sets. Classifiers were trained on the data sets listed across the top and evaluated in the data sets listed across the bottom. Data sets are listed by their GEO accession numbers. Expression (black): gene expression data. WGCNA (blue): module enrichment scores.

**Figure 6:**
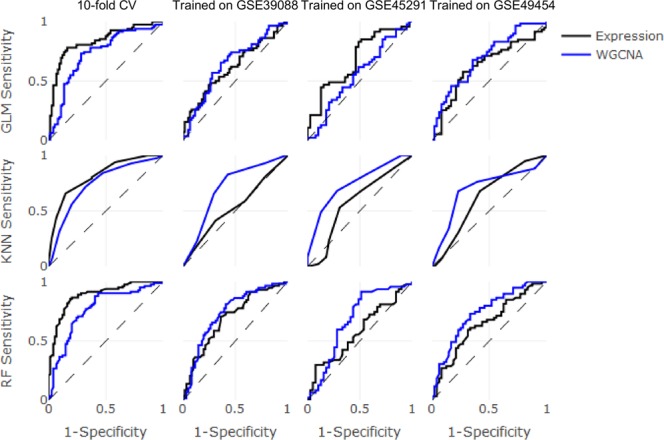
Area under the ROC curve of machine learning classifiers across three independent data sets. Classifiers were trained on the data sets listed across the top and tested in the other two data sets. Data sets are listed by their GEO accession numbers. Expression (black): gene expression data. WGCNA (blue): module enrichment scores.

To assess the effectiveness of either raw gene expression or module-based enrichment techniques, SLE patients were classified as active or inactive using generalized linear models (GLM), *k*-nearest neighbors (KNN), and random forest (RF) classifiers. Classifiers were validated using two different methodologies: (1) 10-fold cross-validation or (2) study-based cross-validation, in which classifiers were trained on each data set independently and tested in the other two data sets. When evaluating the performance of classifiers on the data set on which they were trained, GLM accuracy was defined as one minus the cross-validated classification error from the cv.glmnet() function, and RF accuracy was determined based on out-of-bag predictions. The accuracy of each classifier trained with either gene expression or module enrichment is shown in Fig. 5, and receiver operating characteristic (ROC) curves are plotted in Fig. 6. Classification metrics for each classifier are shown in Table 5.

**Table 5:** Classification metrics of machine learning classifiers.

		10-fold CV		Trained on GSE39088		Trained on GSE45291		Trained on GSE49454	
		Expression	WGCNA	Expression	WGCNA	Expression	WGCNA	Expression	WGCNA
GLM	Accuracy	0.80	0.72	0.51	0.56	0.57	0.56	0.63	0.63
	Sensitivity	0.78	0.73	0.86	0.79	0.51	0.60	0.54	0.59
	Specificity	0.82	0.70	0.18	0.34	0.64	0.51	0.73	0.67
	AUC	0.84	0.73	0.62	0.65	0.68	0.55	0.63	0.69
	Kappa	0.60	0.43	0.04	0.14	0.15	0.11	0.26	0.26
	PPV	0.83	0.73	0.50	0.53	0.63	0.60	0.71	0.69
	NPV	0.77	0.70	0.58	0.64	0.52	0.51	0.56	0.57
KNN	Accuracy	0.75	0.70	0.50	0.70	0.49	0.70	0.51	0.72
	Sensitivity	0.66	0.72	0.59	0.83	0.23	0.68	0.31	0.68
	Specificity	0.85	0.68	0.41	0.57	0.79	0.72	0.77	0.77
	AUC	0.82	0.74	0.54	0.71	0.58	0.75	0.63	0.70
	Kappa	0.50	0.40	0.00	0.40	0.03	0.40	0.07	0.44
	PPV	0.83	0.71	0.49	0.65	0.58	0.74	0.62	0.78
	NPV	0.69	0.68	0.51	0.78	0.46	0.65	0.47	0.66
RF	Accuracy	0.83	0.72	0.45	0.63	0.47	0.63	0.61	0.66
	Sensitivity	0.83	0.77	0.86	0.91	0.53	0.62	0.54	0.61
	Specificity	0.82	0.68	0.07	0.36	0.38	0.64	0.69	0.73
	AUC	0.89	0.77	0.69	0.73	0.58	0.68	0.65	0.74
	Kappa	0.65	0.45	−0.07	0.27	−0.08	0.26	0.22	0.33
	PPV	0.84	0.72	0.47	0.58	0.51	0.67	0.68	0.73
	NPV	0.81	0.72	0.33	0.81	0.41	0.58	0.55	0.60

Training sets are listed by their GEO accession numbers. Test accuracy was determined by testing the classifiers on the other two data sets. Expression: gene expression data. WGCNA: module enrichment scores. AUC: area under the receiver operating characteristic curve. Kappa: Cohen’s kappa coefficient. PPV: positive predictive value. NPV: negative predictive value.

When performing 10-fold cross-validation, the use of gene expression values resulted in better performance from all three classifiers compared to module enrichment scores. The random forest classifier was the strongest performer with 83 percent accuracy, and its corresponding ROC curve demonstrated an excellent tradeoff between recall and fall-out (AUC 0.89). This high accuracy can likely be attributed to the presence of data from all three studies in both the training and test sets. In this case, the classifiers have the opportunity to learn patterns inherent to each data set, which proves useful during testing. To ensure that the classifiers were not disproportionately learning patterns from certain data sets at the expense of others, the classification results from the 10-fold cross-validation approach were subdivided by data set. All classifiers exhibited good performance with small differences between their highest and lowest accuracies in individual data sets, with the exception of the WGCNA-based KNN classifier (Supplementary Table S1).

When performing study-based cross-validation, classifiers trained on expression data performed better on their respective training sets than those trained on module enrichment scores in nearly all cases (Fig. 5). However, the accuracy of classifiers trained on expression values in the test sets was approximately 50 percent. This is in line with the findings of our initial bioinformatic analysis (Table 2), namely, that gene expression values have little to no utility when attempting to classify unfamiliar samples. When the training and test data come from different data sets, the classifiers learn patterns that are unhelpful for classifying test samples. Although classifiers trained on module enrichment scores did not achieve high accuracies in their training sets, they did not experience as sharp a drop in accuracy when tested on unfamiliar data sets. Remarkably, the use of module enrichment scores improved RF test accuracy to approximately 65 percent and improved KNN test accuracy to approximately 70 percent.

Overall, gene expression values provide high accuracy when performing 10-fold cross-validation but are rendered nearly useless when performing study-based cross-validation. These results indicate that disease activity classification based on raw gene expression, while more accurate, is sensitive to technical variability, whereas classification based on module enrichment better copes with variation among data sets.

Random forest consistently achieved high performance, and we hypothesized that its assessments of variable importance could be used to gain insight into directors of the identification of SLE activity. To this end, random forest classifiers were trained on all patients from all data sets in order to identify the most important genes and modules as determined by mean decrease in the Gini impurity, a measure of misclassification error. The classifier trained with gene expression data achieved an out-of-bag accuracy of 81 percent, with a sensitivity of 83 percent and a specificity of 78 percent. The classifier trained with module enrichment scores achieved an out-of-bag accuracy of 73 percent, with a sensitivity of 78 percent and a specificity of 68 percent.

**Figure 7:**
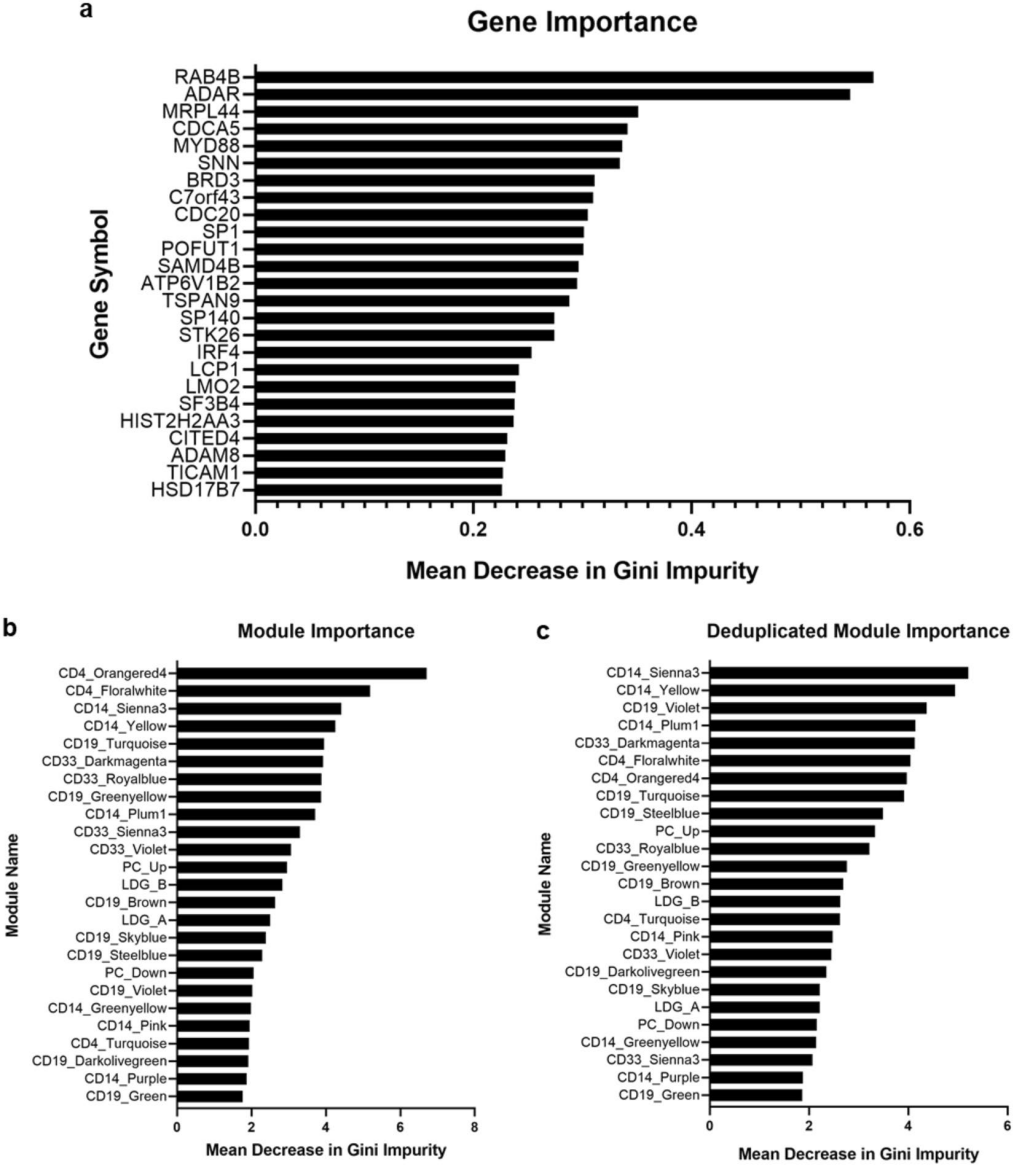
Random forest classifier reveals variable importance of genes and modules. (**a**) Variable importance of top 25 individual genes as determined by mean decrease in Gini impurity. (**b**) Variable importance of cell modules. (**c**) As many modules shared genes, modules were deduplicated to determine the effects on the random forest classifier. The relative importance of the full modules and deduplicated modules was strongly correlated (Spearman’s rho = 0.69, p = 1.94E-4). LDG: low-density granulocyte; PC: plasma cell.

The most important genes and modules identified a wide array of cell types and biological functions (Fig. 7). The most important genes encompass such diverse functions as interferon signaling, pattern recognition receptor signaling, and control of survival and proliferation (Fig. 7a). Notably, the most influential modules skewed away from B cell-derived modules and towards T cell- and myeloid cell-derived modules (Fig. 7b). As some of these modules had overlapping genes, the variable importance experiment was repeated with modules that were first scrubbed of any genes that appeared in more than one module before GSVA enrichment scoring (Supplementary Data S3). The relative variable importance scores of the deduplicated modules correlated strongly with those of the original modules (Spearman’s rho = 0.69, p = 1.94E-4), indicating that module behavior was partly driven by the overlapping genes but strongly driven by unique genes (Fig. 7c).

CD4_Floralwhite and CD14_Yellow, two interferon-related modules which maintained high importance after deduplication, were further analyzed to study the effect of unique genes on module importance. Gene lists were tested for statistical overrepresentation of Gene Ontology biological process terms with FDR correction on pantherdb.org. CD4_Floralwhite did not show any significant enrichment, but CD14_Yellow, which had the highest importance after deduplication, was highly enriched for genes with the “Immune Effector Process” designation (26/77 genes, FDR = 9.38E-11 by Fisher’s exact test). This suggests that CD14+ monocytes express unique genes that may play important roles in the initiation of SLE activity.

## Discussion

Several important findings related to SLE gene expression heterogeneity within and across data sets have been elucidated by this study. First, we demonstrated that DE analysis of active versus inactive patients is insufficient for proper classification of SLE disease activity, as systematic differences between data sets render conventional bioinformatics techniques largely non-generalizable.

Next, we hypothesized that WGCNA modules created from the cellular components of WB and correlated to SLEDAI disease activity might improve classification of disease activity in SLE patients. The use of cell-specific gene modules based on a priori knowledge about their relevance to disease fared slightly better than raw gene expression, as it generated informative enrichment patterns, and many of the modules maintained significant correlations with SLEDAI in WB. However, these enrichment scores failed to separate active patients from inactive patients completely by hierarchical clustering.

We then compared raw expression data alongside the WGCNA generated modules of genes in machine learning applications. We used a supervised classification approach using elastic generalized linear modeling, *k*-nearest neighbors, and random forest classifiers. The trends in performance when cross-validating by study or cross-validating 10-fold speak to the potential advantages and disadvantages of diagnostic tests incorporating gene expression data or module enrichment. Cross-validating by study serves as a kind of “worst-case” scenario, whereas 10-fold cross-validation serves as a “best-case.” Attempting to classify active and inactive SLE patients from different data sets and different microarray platforms during cross-validation by study proved difficult, but module enrichment was able to smooth out much of the technical variation between data sets. 10-fold cross-validation simulated a more standardized diagnostic test. Although the data was sourced from three different microarray platforms, each cohort in the test set had many similar patients in the training set to facilitate classification by gene expression. If such a test could be reliably free from technical noise, it is likely that raw gene expression would perform very well. RNA-Seq platforms, which produce transcript counts rather than probe intensity values, may display less technical variation across data sets because they are not dependent on the binding characteristics of pre-defined probes that differ among arrays^34^. On the other hand, comparison of RNA-Seq and microarray samples has shown that the two methods can deliver highly consistent results^35–37^, so a microarray-based test could be feasible if it was only conducted on one platform. Further study to construct an optimal panel of genes similar to that identified by the random forest classifier could result in a simple, focused test to determine disease activity by gene expression data alone. Interestingly, module enrichment scores, which show little variation across platforms, could be used to develop diagnostic tests that leverage existing data sets, even if they are sourced from different platforms.

The strong performance of the random forest classifier indicates that nonlinear, decision tree-based methods of classification may be best suited to SLE diagnostics. This may be because decision trees ask questions about new samples sequentially and adaptively in contrast to other methods that approach variables from new samples all at once. Random forest is able to “understand” to an extent that different types of patients exist and that a one-size-fits-all approach will tend to misclassify those patients whose expression patterns make them a minority within their phenotype. To put it more simply, active patients that do not resemble the majority of active patients still have a strong chance of being properly classified by random forest.

We used the random forest classifier to assess the importance of each gene and module in patient classification. The most important genes were involved in a number of functions other than interferon signaling, such RNA processing, ubiquitylation, and mitochondrial processes. These pathways may play important roles in directing, or at least be indicative of, SLE disease activity. CD4 T cells originally contributed the most important modules, but when the modules were deduplicated, CD14 monocyte-derived modules gained importance. This suggests that unique genes expressed by CD14 monocytes in tandem with interferon genes may prove to be informative in the study of cell-specific methods of SLE pathogenesis. Futhermore, it is important to note that modules that were negatively associated with disease activity were just as important in classification as positively associated modules. Further study of underrepresented categories of transcripts should enhance our understanding of SLE activity.

One limitation of this study was the relatively small amount of data used to train and test the classifiers. Creating dedicated training and test sets is preferable to cross-validation, but it requires many samples. Although there are large numbers of publicly available gene expression profiles of SLE patients, many of these profiles are not annotated with SLEDAI data. Furthermore, some data sets which include SLEDAI data show heavy class imbalance, which impedes classification. Further work to integrate cross-platform expression data will be crucial to expanding our ability to classify active and inactive SLE patients.

The machine learning models tested here provide the basis of personalized medicine for SLE patients. Integration of our approaches with emerging high-throughput patient sampling technologies could unlock the potential to develop a simple blood test to predict SLE disease activity. Our approaches could also be generalized to predict other SLE manifestations, such as organ involvement. A better understanding of the cellular processes that drive SLE pathogenesis may eventually lead to customized therapeutic strategies based on patients’ unique patterns of cellular activation.

## Methods

### Compilation of gene expression data from SLE patients

Publicly available gene expression data and corresponding phenotypic data were mined from the Gene Expression Omnibus. Raw data sources for purified cell populations are as follows: GSE10325 (CD4: 8 SLE, 9 HC; CD19: 10 SLE, 8 HC; CD33: 9 SLE, 9 HC); GSE26975 (10 SLE LDG, 10 SLE Neutrophil, 9 HC Neutrophil); GSE38351 (CD14: 8 SLE, 12 HC). Raw data sources for SLE whole blood gene expression are as follows: GSE39088 (24 active, 13 inactive); GSE45291 (35 active, 257 inactive); GSE49454 (23 active, 26 inactive). 35 randomly sampled inactive patients were taken from GSE45291 to avoid a major imbalance between active and inactive SLE patients. Active SLE was defined as having an SLE Disease Activity Index (SLEDAI) of 6 or greater.

### Quality control and normalization of raw data files

Statistical analysis was conducted using R and relevant Bioconductor packages. Non-normalized arrays were inspected for visual artifacts or poor hybridization using Affy QC plots. PCA plots were used to inspect the raw data files for outliers. Data sets culled of outliers were cleaned of background noise and normalized using RMA, GCRMA, or NEQC where appropriate. Data sets were then filtered to remove probes with low intensity values and probes without gene annotation data. WB gene expression data sets were filtered to only include genes that passed quality control in all data sets. At this juncture, differential expression (DE) analysis and Weighted Gene Co-expression Network Analysis (WGCNA) were carried out on data sets. WB gene expression data sets were then further processed before machine learning analysis. WB gene expression values were centered and scaled to have zero-mean and unit-variance within each data set, and the standardized expression values from each data set were joined for classification.

### Differential expression analysis

Normalized expression values were variance corrected using local empirical Bayesian shrinkage, and DE was assessed using the LIMMA R package^38^. Resulting p-values were adjusted for multiple hypothesis testing using the Benjamini-Hochberg correction^39^, which resulted in a false discovery rate (FDR). Significant genes within each study were filtered to retain DE genes with an FDR < 0.2, which were considered statistically significant. The FDR was selected a priori to diminish the number of genes that might be excluded as false negatives. Rank-rank hypergeometric overlap between data sets was assessed using the RRHO R package^40^. Additional analyses examined differentially expressed genes with an FDR < 0.05.

### Weighted gene co-expression network analysis (WGCNA) of purified cell populations

Log2-normalized microarray expression values from purified CD4, CD14, CD19, CD33, and low density granulocyte (LDG) populations were used as input to WGCNA to conduct an unsupervised clustering analysis, resulting in co-expression “modules,” or groups of densely interconnected genes which could correspond to comparably regulated biologic pathways^41^. For each experiment, an approximately scale-free topology matrix (TOM) was first calculated to encode the network strength between probes. Probes were clustered into WGCNA modules based on TOM distances. Resultant dendrograms of correlation networks were trimmed to isolate individual modular groups of probes by partitioning around medoids and labeled using color assignments based on module size. Expression profiles of genes within modules were summarized by a module eigengene (ME), which is analogous to the module’s first principal component. MEs act as characteristic expression values for their respective modules and can be correlated with sample traits such as SLEDAI or cell type. This was done by Pearson correlation for continuous or semi-continuous traits and by point-biserial correlation for dichotomous traits.

WGCNA modules from CD4, CD14, CD19, and CD33 cells were tested for correlation to SLEDAI. SLEDAI information was not available for the LDG modules, so the two modules provided are descriptive of LDGs compared to SLE neutrophils and HC neutrophils.

Plasma cell modules were generated by differential expression analysis and not WGCNA, but were included because of the established importance of plasma cells in SLE pathogenesis and their increase in active disease^2^.

### Gene set variation analysis (GSVA)-based enrichment of expression data

The GSVA R package^42^ was used as a non-parametric method for estimating the variation of pre-defined gene sets in SLE WB gene expression data sets. Standardized expression values from WB data sets were used to test for enrichment of cell-specific WGCNA gene modules using the Single-sample Gene Set Enrichment Analysis (ssGSEA) method, which scores single samples in isolation and is thus shielded from technical variation within and among data sets. Statistical analysis of GSVA enrichment scores was done bv Spearman correlation or Welch’s unequal variances *t*-test, where appropriate. Effect sizes were assessed by Cohen’s *d*^43^.

### Machine learning algorithms and parameters

We employed three distinct machine learning algorithms to test biased and unbiased approaches to microarray data analysis. The biased approach involved GSVA enrichment of disease-associated, cell-specific modules, and the unbiased approach employed all available gene expression data in the WB. An elastic generalized linear model (GLM), k-nearest neighbors classifier (KNN), and random forest (RF) classifier were deployed to classify active and inactive SLE patients and determine whether gene expression could serve as a general predictor of disease activity. GLM, KNN, and RF were deployed using the glmnet, caret, and randomForest R packages, respectively^44–46^.

GLM carries out logistic regression with a tunable elastic penalty term to find a balance between the L1 (lasso) and L2 (ridge) penalties and thereby facilitate variable selection. For our predictions, the elastic penalty was set to 0.9, specifying a penalty that is 90% lasso and 10% ridge in order to generate sparse solutions. KNN classifies unknown samples based on their proximity to a set number *k* of known samples. *K* was set to 5% of the size of the training set. If the initial value of *k* was even, 1 was added in order to avoid ties. RF generates 500 decision trees which vote on the class of each sample. The Gini impurity index, a measure of misclassification error, was used to evaluate the importance of variables^47^.

### Validation approaches

The performance of each machine learning algorithm was evaluated by 2 different forms of cross-validation. First, a random 10-fold cross-validation was carried out by randomly assigning each patient to one of 10 groups. For each pass of cross-validation, one group was held out as a test set, and the classifiers were trained on the remaining data. Next, as the data came from three separate studies, study-based cross-validation was also done to determine the effects of systematic technical differences among data sets on classification performance. In this circumstance, the classifiers were trained on one data set and tested in the other two data sets. Accuracy was assessed as the proportion of patients correctly classified across all testing folds. Performance metrics such as sensitivity and specificity were assessed after cross-validation by agglomerating class probabilities and assignments from each fold or study. Receiver Operating Characteristic (ROC) curves were generated using the pROC R package^48^.

## Supplementary information

Supplementary Information

Dataset 1

Dataset 2

Dataset 3

## Data Availability

All data sets used in these analyses may be downloaded from GEO using the accession numbers provided in the methods.
