# Supplementary material for: Machine learning approaches to predict lupus disease activity from gene expression data
Source: Sci Rep. 2019 Jul 3;9:9617. doi: 10.1038/s41598-019-45989-0 (PMC6610624; doi:10.1038/s41598-019-45989-0)
Supplement: Supplementary file 1 — Supplementary Information [file 41598_2019_45989_MOESM1_ESM.pdf]

Supplementary Information

Machine learning approaches to predict lupus disease activity from gene expression data

Brian Kegerreis[1], Michelle D. Catalina[1], Prathyusha Bachali[1], Nicholas S. Geraci[1], Adam C. Labonte[1], Chen Zeng[2], Nathaniel Stearrett[3], Keith A. Crandall[3], Peter E. Lipsky[1] and Amrie C. Grammer[1]*.

1 RILITE Research Institute and AMPEL BioSolutions, 250 W Main St, Ste 300, Charlottesville, VA 22902
2 Department of Physics, George Washington University, Washington DC 20052
3 Computational Biology Institute, Milken Institute School of Public Health, George Washington University, Washington DC 20052

*corresponding author: amriegrammer@comcast.net

Supplementary Table S1

		Subset: GSE39088		Subset: GSE45291		Subset: GSE49454		Range	
		Expression	WGCNA	Expression	WGCNA	Expression	WGCNA	Expression	WGCNA
GLM	Accuracy	0.81	0.70	0.83	0.74	0.76	0.69	0.07	0.05
	Sensitivity	0.73	0.73	0.83	0.71	0.76	0.76	0.10	0.05
	Specificity	0.93	0.67	0.83	0.77	0.75	0.63	0.18	0.14
	AUC	0.85	0.74	0.84	0.75	0.84	0.70	0.01	0.05
	Kappa	0.63	0.39	0.66	0.49	0.51	0.39	0.15	0.10
	PPV	0.94	0.76	0.83	0.76	0.76	0.68	0.18	0.08
	NPV	0.70	0.63	0.83	0.73	0.75	0.71	0.13	0.10
KNN	Accuracy	0.78	0.84	0.76	0.70	0.71	0.59	0.07	0.25
	Sensitivity	0.68	0.86	0.71	0.71	0.56	0.60	0.15	0.26
	Specificity	0.93	0.80	0.80	0.69	0.88	0.58	0.13	0.22
	AUC	0.85	0.84	0.79	0.75	0.84	0.65	0.06	0.19
	Kappa	0.58	0.66	0.51	0.40	0.43	0.18	0.15	0.48
	PPV	0.94	0.86	0.78	0.69	0.83	0.60	0.16	0.26
	NPV	0.67	0.80	0.74	0.71	0.66	0.58	0.08	0.22
RF	Accuracy	0.81	0.81	0.83	0.71	0.84	0.67	0.03	0.14
	Sensitivity	0.82	0.82	0.86	0.74	0.80	0.76	0.06	0.08
	Specificity	0.80	0.80	0.80	0.69	0.88	0.58	0.08	0.22
	AUC	0.87	0.86	0.90	0.78	0.88	0.72	0.03	0.14
	Kappa	0.61	0.61	0.66	0.43	0.67	0.34	0.06	0.27
	PPV	0.86	0.86	0.81	0.70	0.87	0.66	0.06	0.20
	NPV	0.75	0.75	0.85	0.73	0.81	0.70	0.10	0.05

Supplementary Table S1. Classification metrics of 10-fold CV machine learning classifiers with results subdivided by data set. Data sets are listed by their GEO accession numbers. Range: difference between maximum and minimum values for each metric. Expression: gene expression data. WGCNA: module enrichment scores. AUC: area under the receiver operating characteristic curve. Kappa: Cohen's kappa coefficient. PPV: positive predictive value. NPV: negative predictive value.

Supplementary Data S1. Differential expression results for active versus inactive lupus patients in three data sets

Supplementary Data S2. WGCNA module gene lists

Supplementary Data S3. WGCNA module gene lists with unique genes only
